# Oncogenic transformation of NIH/3T3 cells by the overexpression of L-type amino acid transporter 1, a promising anti-cancer target

**DOI:** 10.18632/oncotarget.27981

**Published:** 2021-06-22

**Authors:** Natsumi Hayashi, Akitaka Yamasaki, Shiho Ueda, Shogo Okazaki, Yoshiya Ohno, Toshiyuki Tanaka, Yuichi Endo, Yoshihisa Tomioka, Kazue Masuko, Takashi Masuko, Reiko Sugiura

**Affiliations:** ^1^Laboratory of Molecular Pharmacogenomics, Faculty of Pharmacy, Kindai University, Higashiosaka-Shi, Osaka, Japan; ^2^Cell Biology Laboratory, School of Pharmacy, Kindai University, Osaka, Japan; ^3^Laboratory of Oncology Pharmacy Practice and Science, Graduate School of Pharmaceutical Sciences, Tohoku University, Sendai-Shi, Miyagi, Japan; ^4^Division of Cell Fate Regulation, Research Institute for Biomedical Sciences, Tokyo University of Science, Noda-shi, Chiba, Japan; ^5^Laboratory of Immunobiology, Department of Pharmacy, School of Pharmacy, Hyogo University of Health Sciences, Kobe-Shi, Hyogo, Japan; ^6^Natural Drug Resources, Faculty of Pharmacy, Kindai University, Osaka, Japan; ^*^Co-first authors; ^#^This laboratory (April, 2000~) was closed at the end of March, 2020, after the mandatory retirement of Takashi Masuko

**Keywords:** CD98, LAT1, monoclonal antibody, NIH/3T3, oncogenicity

## Abstract

L-type amino acid transporter 1 (LAT1)/SLC7A5 is the first identified CD98 light chain disulfide linked to the CD98 heavy chain (CD98hc/SLC3A2). LAT1 transports large neutral amino acids, including leucine, which activates mTOR, and is highly expressed in human cancers. We investigated the oncogenicity of human LAT1 introduced to NIH/3T3 cells by retrovirus infection. NIH/3T3 cell lines stably expressing human native (164C) or mutant (164S) LAT1 (naLAT1/3T3 or muLAT1/3T3, respectively) were established. We confirmed that endogenous mouse CD98hc forms a disulfide bond with exogenous human LAT1 in naLAT1/3T3, but not in muLAT1/3T3. Endogenous mouse CD98hc mRNA increased in both naNIH/3T3 and muLAT1/3T3, and a similar amount of exogenous human LAT1 protein was detected in both cell lines. Furthermore, naLAT1/3T3 and muLAT1/3T3 cell lines were evaluated for cell growth-related phenotypes (phosphorylation of ERK, cell-cycle progression) and cell malignancy-related phenotypes (anchorage-independent cell growth, tumor formation in nude mice). naLAT1/3T3 had stronger growth- and malignancy- related phenotypes than NIH/3T3 and muLAT1/3T3, suggesting the oncogenicity of native LAT1 through its interaction with CD98hc. Anti-LAT1 monoclonal antibodies significantly inhibited *in vitro* cell proliferation and *in vivo* tumor growth of naLAT1/3T3 cells in nude mice, demonstrating LAT1 to be a promising anti-cancer target.

## INTRODUCTION

CD98 was originally reported as a cell-surface marker associated with lymphocyte activation [1], and subsequently identified as a unique molecule expressed by numerous cancer cells [2–4]. CD98 with a molecular weight (MW) of 125 kDa / gp125 [2, 3] is composed of a CD98 heavy chain (CD98hc) with a MW of 80~100 kDa and CD98 light chains (CD98lcs) with a MW of 35~55 kDa [1–4]. CD98hc, also referred to as solute carrier (SLC) 3A2, can bind to six CD98lcs of the SLC7A amino acid transporter family (SLC7A5~7A8, 7A10, and 7A11) [5–11]. SLC7A11 (xCT) was identified as a molecule required for the maintenance of cancer stem cells (CSCs) [12]. Variant form of CD44 (CD44v) [12–17] and HER1 [18] associate with xCT in epithelial cancers or gliomas and stabilize xCT, respectively, resulting in the survival of CSCs in the oxidative stress by anti-cancer drugs. In this context, we demonstrated the anti-tumor effects of fully-human mAb [19] recognizing CD44v bound to xCT expressed by CSCs.

L-type amino acid transporter 1 (LAT1)/SLC7A5 is the first identified CD98lc disulfide-linked to CD98hc. LAT1 transports large neutral amino acids, including branched-chain amino acids (BCAA), and is highly expressed in human cancers irrespective of the tissue origin.

The NIH/3T3 transformation system [20] led to the discovery of many oncogene-encoded oncoproteins, including RAS family proteins, human epidermal growth factor receptor 1 (HER1/EGFR) and HER2. HER1 and HER2 are overexpressed in numerous human cancers and became suitable targets for cancer therapy using low-molecular-weight compounds or monoclonal antibodies (mAbs). We previously demonstrated the oncogenicity of CD98hc in the NIH/3T3 and Balb/3T3 transformation systems [21–23]. In addition, NIH/3T3 cells over-expressing CD98hc resist early G1 arrest and apoptosis induced by serum starvation [24]. The oncogenicity of CD98 was confirmed with wild-type CD98hc [21–23] bound to CD98lc, but not with mutant CD98hc [23] lacking the cysteine residue needed for the association with CD98lcs. This suggests that molecular complexes between CD98hc and some CD98lcs are oncogenic, and we identified CD98hc-LAT1 as an oncogenic complex by targeted disruption of the chicken LAT1 gene [25]. Although the major targets of existing anti-cancer therapeutic antibodies are receptor-type tyrosine kinases, overexpression of HER1 and HER2 is limited to cancers from squamous [26, 27] or glandular [28, 29] epithelium, respectively. As CD98/LAT1 is expressed by almost all cancers irrespective of tissue origin [2, 3, 30–40], therapeutic antibodies may be ideal against numerous human malignancies. Although clinical trials with anti-CD98hc antibodies were recently started [41], the cancer specificity of LAT1 is superior to that of CD98hc [42–44].

In this study, we thoroughly examined the oncogenicity of native and mutant human LAT1 in the NIH/3T3 system, followed by several biological analyses using NIH/3T3 cell lines expressing native or mutant LAT1, and found LAT1 to be a promising anti-cancer target.

## RESULTS

### Establishment of NIH/3T3 cell lines expressing native or mutant human LAT1

**Figure 1:**
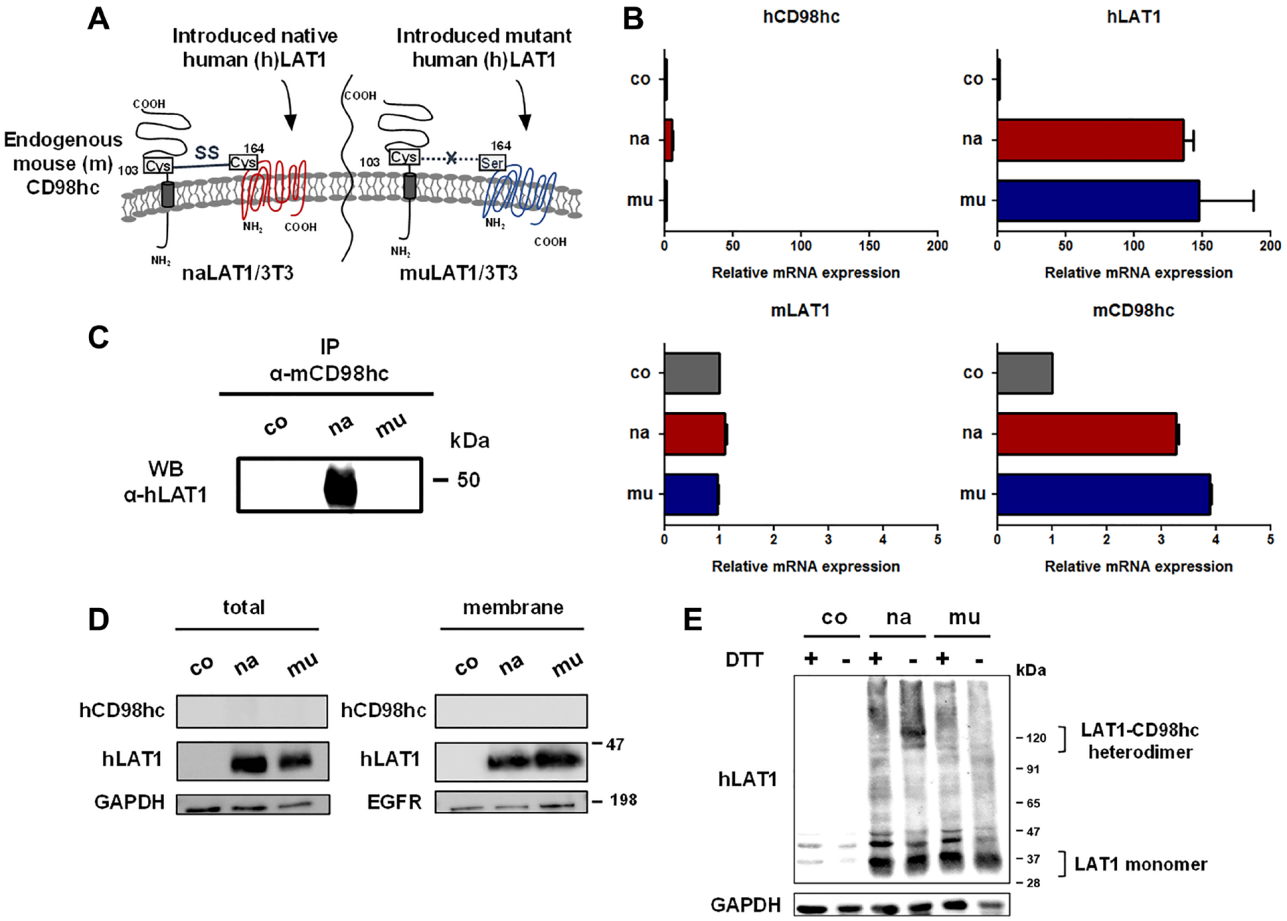
Establishment and basic characterization of NIH/3T3 cell lines expressing human native or mutant LAT1 protein. (**A**) Schematic illustration of the cell surface of NIH/3T3 cell lines expressing human native or mutant LAT1. (**B**) RT-qPCR analysis of human and mouse LAT1/CD98hc expression in NIH/3T3 cell lines. (**C**) Association of exogenously expressed human naLAT1, but not muLAT1, with endogenous mouse CD98hc. Cell lysates from NIH/3T3 cell lines were subjected to IP with anti-mouse CD98hc mAb and to WB with anti-human LAT1 rabbit pAb. (**D**) Protein expression of human LAT1/CD98hc in whole lysates or membrane fraction was analyzed by WB. Blots of hCD98hc were used as the negative control. (**E**) WB analysis of LAT1 protein under reducing or non-reducing conditions using NIH/3T3 cell lines. Lysates from NIH/3T3 cell lines were subjected to SDS-PAGE in under reducing or non-reducing conditions, and to WB with anti-LAT1 pAb.

We established NIH/3T3 cell lines infected with a vacant pMYs-IRES-Puro retrovirus, or the same retrovirus containing cDNA encoding human (h) native (164C) LAT1 or mutant (164 S) LAT1, whose 164th cysteine is genetically converted to serine. The infected cells were selected by puromycin, and designated as co (coNIH/3T3: puromycin-resistant control NIH/3T3), na (naLAT1/3T3: NIH/3T3 expressing native LAT1), and mu (muLAT1/3T3: NIH/3T3 expressing mutant LAT1). The schematic for the NIH/3T3 cell lines expressing native and mutant LAT1 is depicted in Figure 1A. The relative mRNA expression of introduced (exogenous) hLAT1, and endogenous mouse (m) LAT1 and CD98hc in the NIH/3T3 cell lines was examined (Figure 1B). The level of exogenous hLAT1 mRNA expression was indistinguishable between naLAT1/3T3 and muLAT1/3T3 cells. Regarding endogenous mouse LAT1 and CD98hc, the CD98hc mRNA level increased in both naLAT1/3T3 and muLAT1/3T3 compared with coNIH/3T3 cells, although mLAT1 mRNA levels were almost equivalent among the three NIH/3T3 cell lines. The possible association of exogenous hLAT1 proteins with endogenous mCD98hc proteins was examined by immunoprecipitation (IP) using anti-mouse CD98hc rat mAb followed by western blot (WB) with anti-human LAT1 rabbit pAb. The association of exogenously expressed naLAT1, but not muLAT1, with endogenous mCD98hc was demonstrated (Figure 1C), supporting that 164C of exogenous hLAT1 forms a disulfide bond with 103C of endogenous mCD98hc. Based on analysis of exogenous hLAT1 proteins, similar amounts of naLAT1 and muLAT1 proteins were expressed, and both naLAT1 and muLAT1 proteins existed in membrane fractions, possibly embedded in the endoplasmic or the plasma membranes (Figure 1D). As shown in Figure 1E, approximately 40-kDa LAT1 proteins were detected both in naLAT1/3T3 and muLAT1/3T3 cells under reducing conditions, and the complexed LAT1-CD98 protein (125 kDa) was detected only in naLAT1/3T3 under non-reducing conditions. Of note, 40-kDa LAT1 was also detected in naLAT1/3T3 cell under non-reducing conditions, suggesting that the association of the disulfide bond between CD98hc and LAT1 is not necessarily required for the membrane trafficking of LAT1, and that LAT1 monomers in addition to LAT1-CD98hc heterodimers exist on the cell surface.

### Phenotypes of NIH/3T3 cells overexpressing LAT1 in the monolayer culture

**Figure 2:**
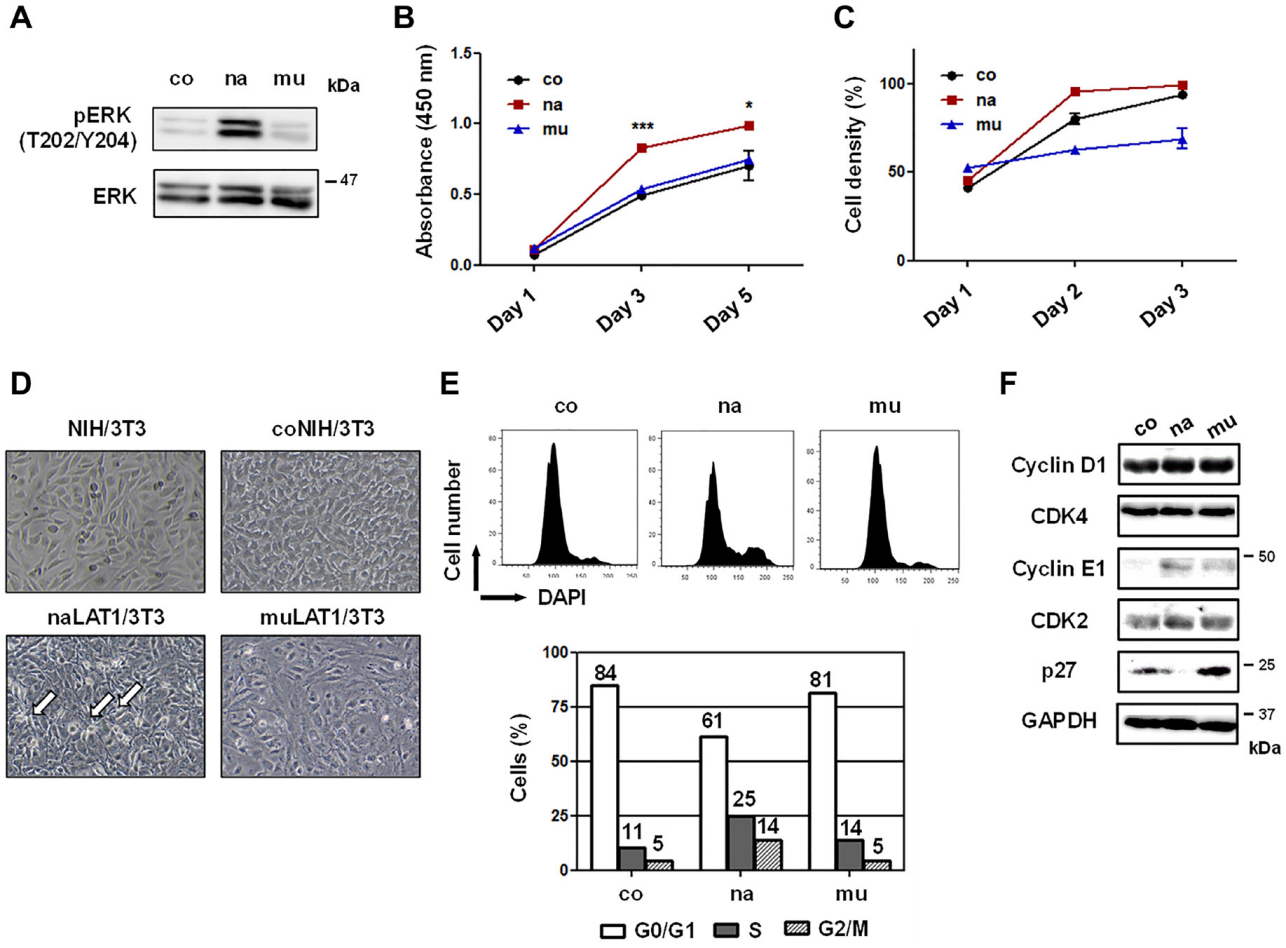
Cell growth-related phenotypes of NIH/3T3 cell lines overexpressing human LAT1 proteins. (**A**) Total and phosphorylated (p-) ERK protein levels were measured by WB. (**B**) Cell growth of NIH/3T3 cell lines cultured in 96-well plates was evaluated by the WST-8 assay. (**C**) Cell density of monolayer cells cultured in 6-well plates was periodically determined by a microscope with CKX-CCSW software. (**D**) Images of the cell morphology in near-confluent monolayers were acquired by CKX53 microscope with a DP22-CUSW digital camera. Arrows show crisscrossed regions. (**E**) Cell-cycle progression was analyzed using ethanol-fixed and DAPI-stained cells by FCM. (**F**) Cell-cycle regulatory proteins were stained with rabbit pAbs and analyzed by WB.

The phosphor- (p-) ERK level in naLAT1/3T3 cells was higher than that in coNIH/3T3 or muLAT1/3T3 cells (Figure 2A). As for cellular growth, naLAT1/3T3 cells grew faster than coNIH/3T3 or muLAT1/3T3 cells (Figure 2B). Regarding the saturation density, the greatest was naLAT1/3T3, followed by coNIH/3T3 and muLAT1/3T3 (Figure 2C). In this context, loss of contact inhibition and high saturation density are well-known indices for transformed or malignant cells. The morphology of these cells in the near confluent state is depicted in Figure 2D. In naLAT1/3T3 cells, a high-density status with local crisscrossed regions (indicated by arrows), which consisted of spindle-shaped cells, was observed, although coNIH/3T3 and muLAT1/3T3 cells exhibited a well-aligned status with contact inhibition, as observed in the wild-type NIH/3T3 cells. As naLAT1/3T3 cells demonstrated accelerated cell growth and increased p-ERK expression, we next analyzed cell-cycle progression by FCM (Figure 2E) and examined cell-cycle regulatory proteins by WB (Figure 2F). Although approximately 15% of control and muLAT1 transfectant cells were in S and G2/M phases, approximately 25% of naLAT1/3T3 cells entered into S phase and approximately 40% were in S and G2/M phases (Figure 2E), supporting the accelerated cell growth of naLAT1/3T3 cells shown in Figure 2B. Increased expression of cyclin E1 in naLAT1/3T3 and muLAT1/3T3 cells compared with coNIH/3T3, and decreased expression of p27 in only naLAT1/3T3 cells were noted (Figure 2F).

### Colony and tumor formation of NIH/3T3 cells overexpressing LAT1

**Figure 3:**
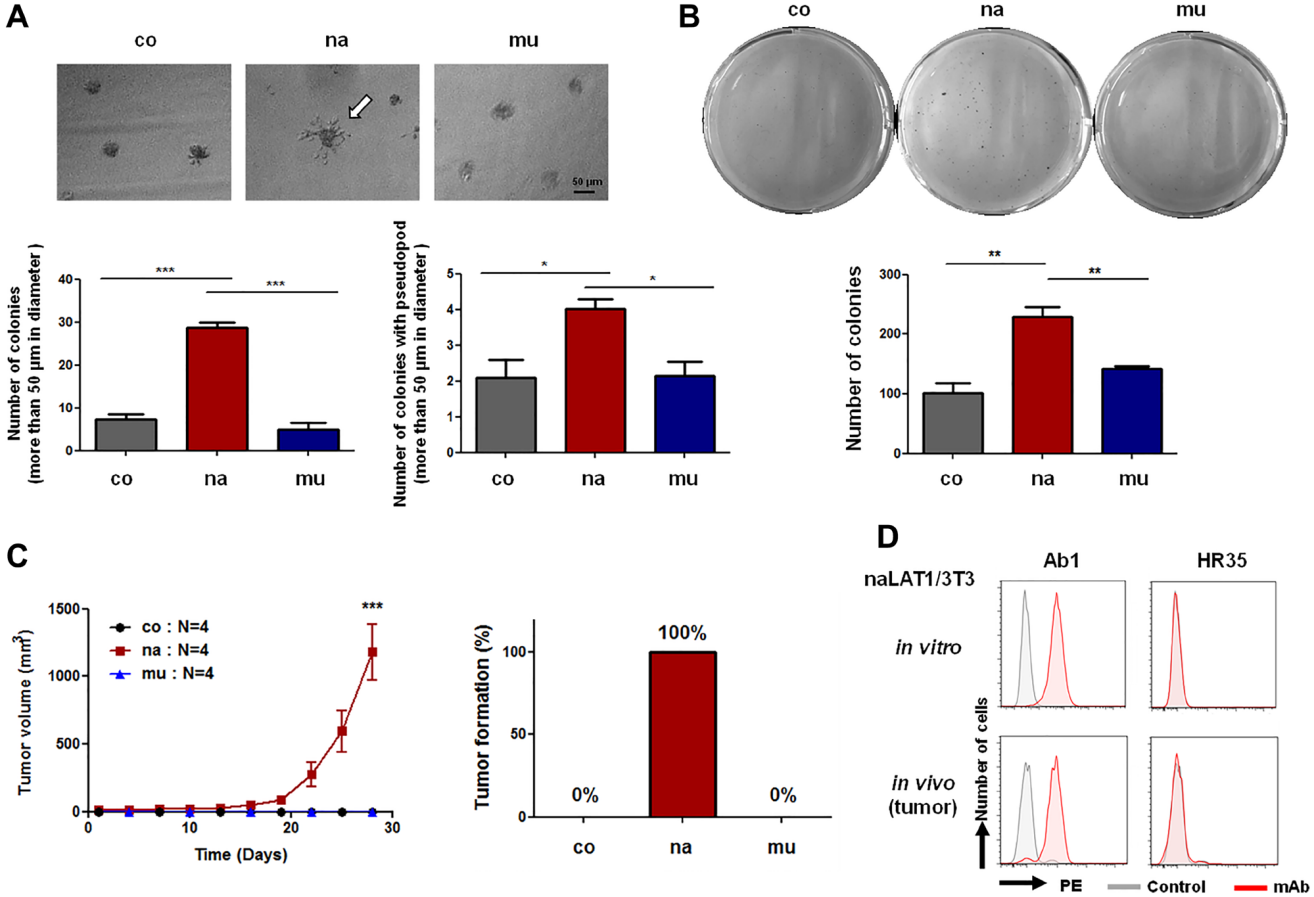
Cell malignancy-related phenotypes of NIH3T3 cell lines overexpressing human LAT1 proteins. (**A**) Anchorage-dependent growth in Matrigel. After the cell culture for 7 days, the number of colonies larger than 50 μm (Lower left) and colonies with pseudopods (Lower right) was counted. The white arrow indicates a colony with pseudopod (Upper). (**B**) Anchorage-independent growth in 3D culture. Cells (2.5 × 10^2^) in 6-well plates were cultured for 14 days in soft agar, stained with Giemsa solution, and the number of colonies was analyzed by Image J. Statistical analysis was carried out using one-way ANOVA. (**C**) Tumor formation by NIH/3T3 cell lines overexpressing LAT1. Tumor volumes were measured every two days. (**D**) FCM using dispersed cell suspension from *in vitro* cultured and *in vivo* (tumor-derived) naLAT1/3T3 was carried out. Ab1, anti-human LAT1 mAb; HR35, anti-human CD98hc mAb.

The anchorage-dependent colony-forming efficiency of naLAT1/3T3 cells was much higher than that of coNIH/3T3 and muLAT1/3T3 cells (Figure 3A, lower left). In addition, an increase in the number of pseudopods of colonies in naLAT1/3T3 cells was observed (Figure 3A, upper and lower right). Next, we investigated the effects of naLAT1 or muLAT1 overexpression on anchorage-independent growth of NIH/3T3 cells in soft-agar medium. The colony forming efficiency of naLAT1/3T3 cells was much higher than that of control coNIH/3T3 and muLAT1/3T3 cells (Figure 3B, upper dish images and lower bar graph)

Next, we evaluated the tumor-formation ability of NIH/3T3 cell lines in nude mice. Five days after cells were inoculated, tumors derived from naLAT1/3T3 cells were confirmed and were allowed to develop until the ethical endpoint in the experimental protocol (Figure 3C). On the other hand, tumors did not develop in all mice transplanted with control coNIH/3T3 or muLAT1/3T3. To analyze the cell population in developed tumors, dispersed tumor cells were evaluated for reactivity to anti-LAT1 mAb by FCM. Anti-LAT1 mAb reacted against tumor-derived (*in vivo*) cells, as in the case of successively cultured (*in vitro*) naLAT1/3T3 (Figure 3D).

### Effects of anti-LAT1 mAb on NIH/3T3 cells overexpressing LAT1

**Figure 4:**
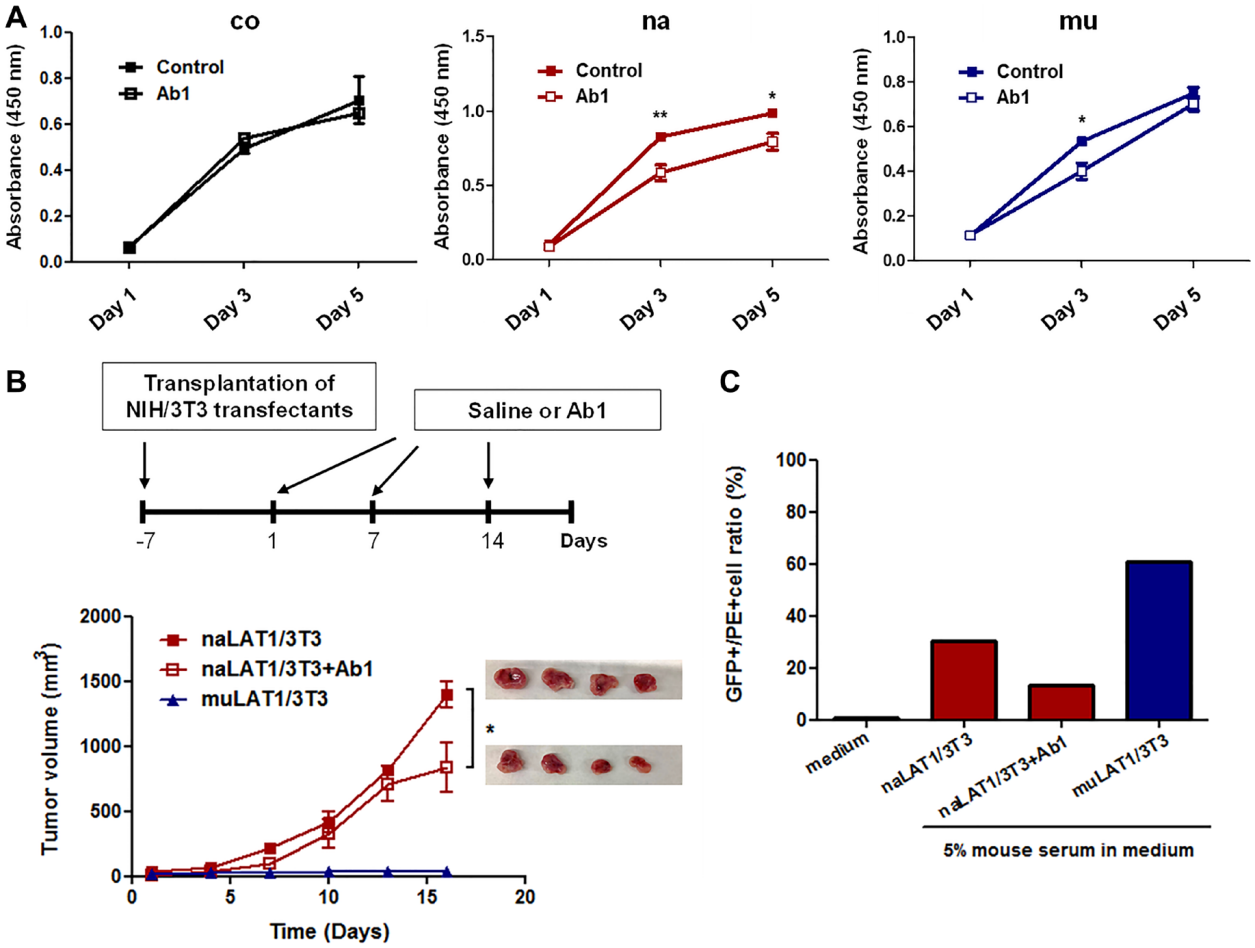
Effects of anti-LAT1 (Ab1) mAb on NIH/3T3 cell lines overexpressing LAT1, and mouse antibody production against NIH/3T3 cell lines expressing native or mutant human LAT1. (**A**) Effects of Ab1 on the *in vitro* cellular growth of NIH3T3 cell lines. Data are shown as the mean ± SEM, and statistical analysis was carried out using one-way ANOVA followed by Tukey’s post hoc multiple comparison test. (**B**) Effects of Ab1 on the *in vivo* tumor growth of naLAT1/3T3 cells. After visible tumors were confirmed (day 0), Ab1 (100 μg/mouse) was intraperitoneally injected on days 1 and 7. (**C**) Production of anti-LAT1 mouse antibodies in nude mice inoculated with NIH/3T3 cell lines. The serum anti-human LAT1 level was analyzed by the reactivity against HEK293 cells expressing human LAT1 fused to GFP by FCM.

Anti-LAT1 mAb (Ab1) significantly inhibited the *in vitro* cellular growth of naLAT1/3T3 cells, although the growth of human LAT1-negative coNIH/3T3 cells was not affected (Figure 4A). Although Ab1 modestly inhibited the growth of muLAT1/3T3 on Day 3, the sensitivity of muLAT1/3T3 to Ab1 was lower than that of naLAT1/3T3, and Ab1 barely inhibited the growth of muLAT1/3T3 on Day 5 (Figure 4A). Next, we examined the *in vivo* effects of Ab1 on the growth of naLAT1/3T3 cells in nude mice (Figure 4B). Tumor growth by naLAT1/3T3 was significantly inhibited by the systemic administration of Ab1, although tumor formation of muLAT1 was again not observed (Figure 4B).

To analyze possible antibody production against human LAT1 protein in nude mice, mouse sera were evaluated by FCM for binding to HEK293 cells expressing human LAT1 fused to GFP. The reactivity of mouse antibodies against human LAT1-GFP was high in muLAT1/3T3, compared with naLAT1/3T3 (Figure 4C). The level of mouse anti-human LAT1 decreased in Ab1-treated mice inoculated with naLAT1/3T3 cells, suggesting that binding of anti-human LAT1 mouse antibodies was at least in part competitively inhibited by Ab1.

### High-affinity binding of anti-LAT1 mAb against muLAT1/3T3

**Figure 5:**
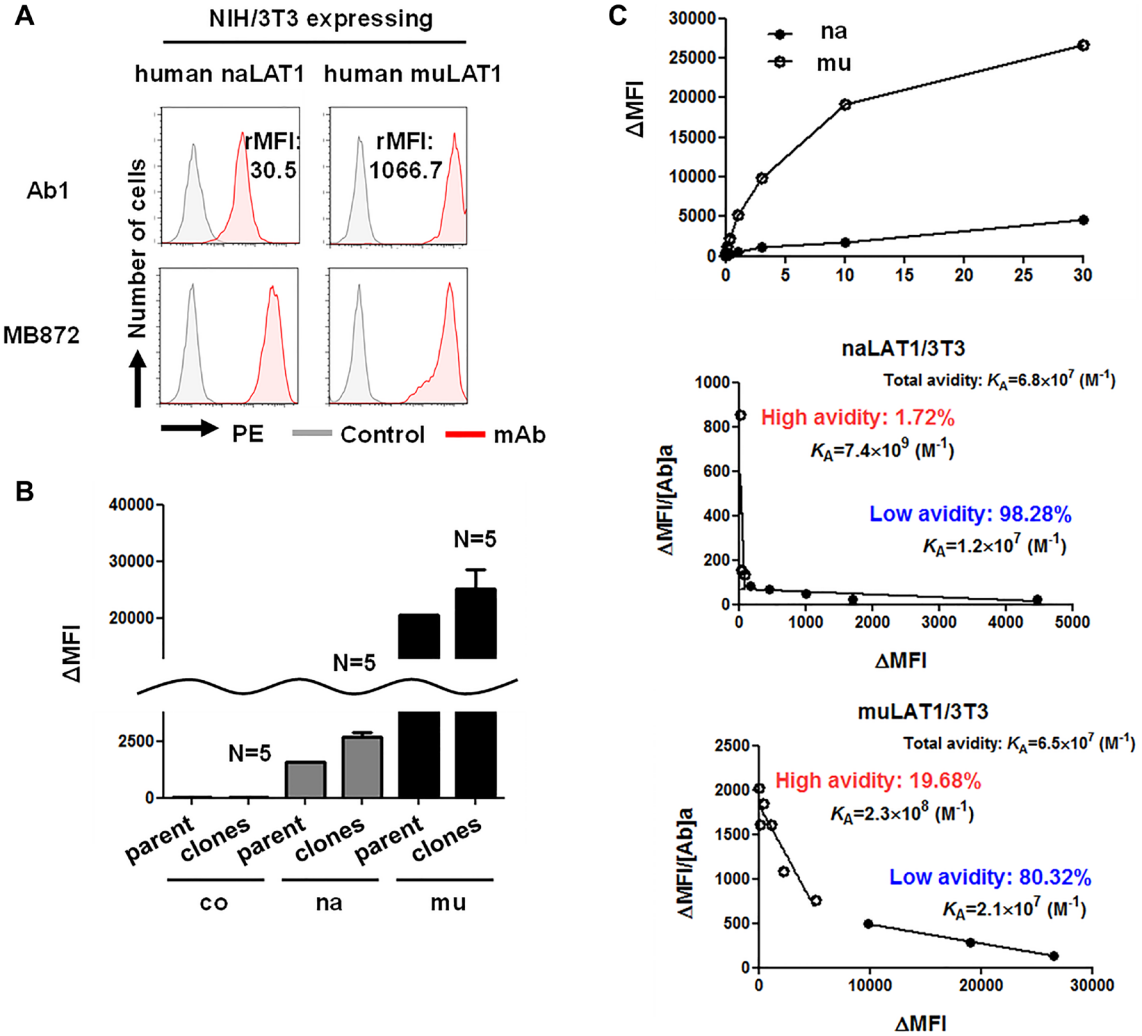
High-affinity binding of anti-LAT1 mAb against muLAT1/3T3 and CD98hc-independent cell-surface expression of LAT1 in human cells. (**A**) Reactivity of anti-human LAT1 mAb against NIH/3T3 cell lines expressing native or mutant human LAT1. From the values of MFI with or without the primary mAbs, the subtracted (Δ) MFI or the ratio (+ mAb / − mAb) of MFI (rMFI) was calculated. (**B**) Reactivity of anti-LAT1 mAb against multiple cell clones from coNIH/3T3, naLAT1/3T3, and muLAT1/3T3 cells. The reactivity of Ab1 was compared by the ΔMFI values. (**C**) Maximum binding activity (left) and binding avidity (middle and right) of anti-human LAT1 rat mAb (Ab1). In the Scatchard plot analysis with naLAT1/3T3 (middle) and muLAT1/3T3 (right) cell lines, cells were reacted with Ab1 at the indicated concentrations (1 ng/mL ~ 30 μg/mL) and analyzed by FCM. The ΔMFI/mAb conc (vertical axis) was plotted against ΔMFI (horizontal axis), and K_A_ (M^-1^) values were determined from the slope of linear regression.

The expression of human LAT1 mRNA (Figure 1B) and protein (Figure 1D) was almost equivalent in both naLAT1/3T3 and muLAT1/3T3 cells. We evaluated the binding of anti-human LAT1 rat mAb (Ab1) to naLAT1/3T3 and muLAT1/3T3 cells. Of note, the reactivity of Ab1 against muLAT1/3T3 was stronger than that against naLAT1/3T3 according to rMFI (1066.7 versus 30.5: approximately 30-fold intensity) analyzed by flow cytometry (FCM) (Figure 5A). This difference was not considered to be caused by the expression level of human LAT1 proteins, as shown in Figure 1D. Comparative analysis using multiple cell clones from coNIH/3T3, naLAT1/3T3, and muLAT1/3T3 cells also produced the same results (Figure 5B), disproving the hypothesis that the difference in LAT1 expression resulted from incidental higher expression of mutant LAT1 during the process of cell line establishment. To analyze the binding characteristics of anti-LAT1 mAb against native and mutant LAT1 proteins in the cell surface in more detail, Scatchard plot analysis [44] with naLAT1/3T3 and muLAT1/3T3 cells was carried out (Figure 5C). Ab1 demonstrated dual avidity (high: 7.4×10^9^ M^-1^, low: 1.2×10^7^ M^-1^) modes in naLAT1/3T3 cells, and exhibited avidity (high: 2.3×10^8^ M^-1^, low: 2.1×10^7^ M^-1^) in muLAT1/3T3 cells, suggesting that the high-avidity binding of muLAT1/3T3 (19.68%) with anti-LAT1 mAb played a role in the large ΔMFI value compared with naLAT1/3T3 (1.72%).

### TCGA analyses (expression, metastasis and prognosis) and possible CD98hc-independent cell-surface expression of LAT1 in human cells

**Figure 6:**
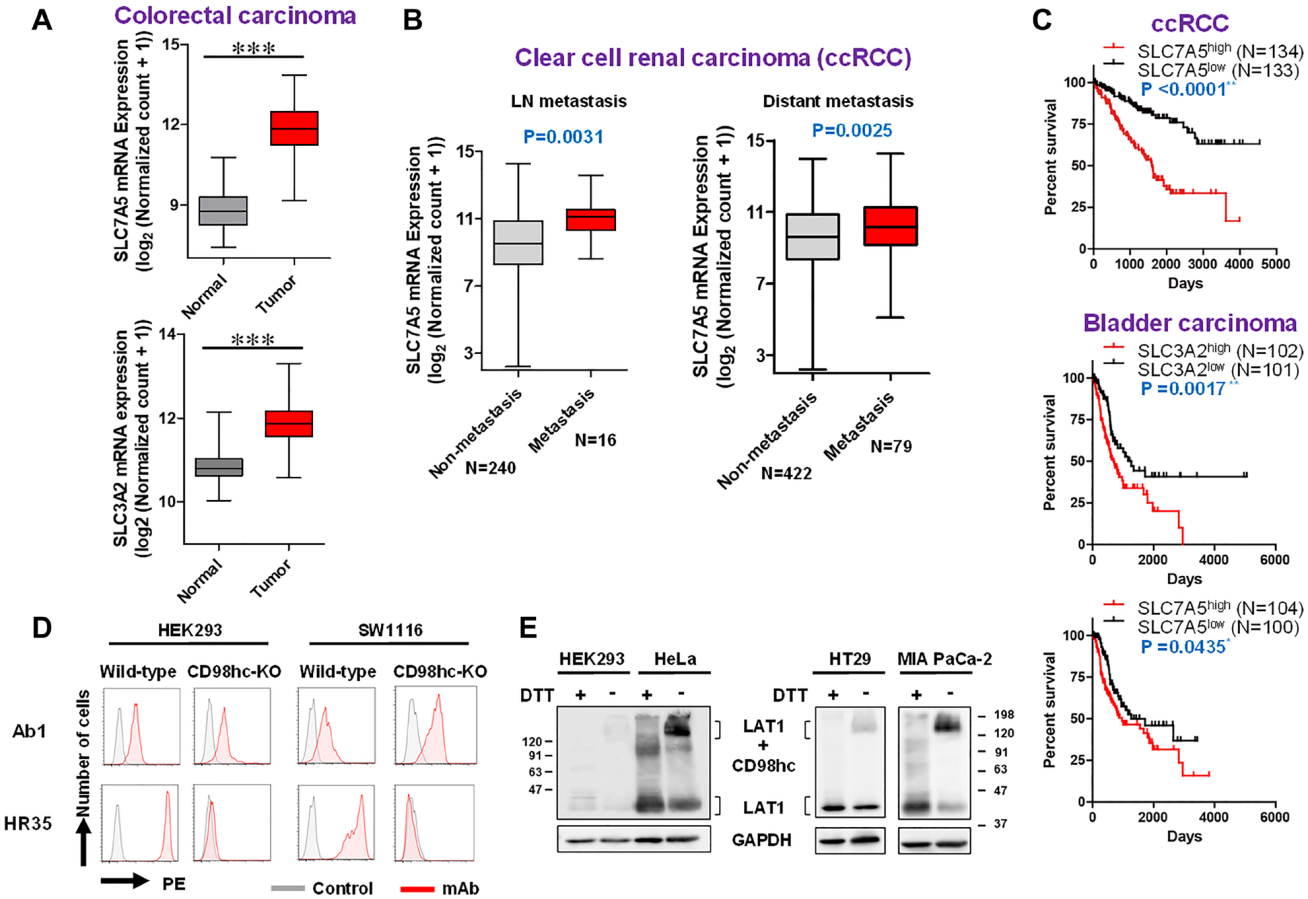
TCGA analyses (expression, metastasis and prognosis) and possible CD98hc-independent cell-surface expression of LAT1 in human cells. TCGA data (**A**–**C**) were obtained from the UCSC Xena browser. (A) Box-whisker plot of the expression of CD98hc (right) /LAT1 (left) in normal and tumor colorectal tissues. *P*-values were calculated by the Student’s *t* test. (B) Box-whisker plot of the expression of LAT1 in non-metastatic and metastatic ccRCC. The presence of metastasis was determined based on pathological TMN classification. *P*-values were calculated by the Student’s *t* test. (C) Kaplan-Meier survival analysis with the log rank test of ccRCC and bladder cancer patient. The correlation between mRNA expression (top 25% and lower 25%) and survival was evaluated. (**D**) Effects of CD98hc-KO on the expression of LAT1. CD98hc in HE293F and SW1116 cells was disrupted by the CRISPR/Cas9-based method, and the expression of CD98hc and LAT1 was analyzed by FCM. (**E**) WB analysis of LAT1 protein under reducing or non-reducing conditions using various human cell lines. Lysates were subjected to SDS-PAGE under reducing or non-reducing conditions, and to WB with anti-LAT1 rabbit pAb.

Based on analyses with The Cancer Genome Atlas (TCGA), overexpression of LAT1/CD98hc was marked in human colorectal cancers, as compared with normal human colorectal tissues (Figure 6A). Coordinated overexpression of both LAT1 and CD98hc was observed in human cancers originating from the esophagus, head and neck, lung and breast (Supplementary Figure 1). Overexpression of LAT1, but not CD98hc, was detected in uterine cervical and bile duct carcinomas, and that of CD98hc, but not LAT1, was noted in glioblastoma, bladder carcinoma, esophagus adenocarcinoma, and Chronophobe renal carcinoma (chRCC) (Supplementary Figure 2). The correlation between higher LAT1 expression and LN/distant metastases of clear cell renal carcinoma (ccRCC) was confirmed (Figure 6B). The overall survival of ccRCC patients with high-level mRNA expression (top 25%) was markedly lower than that of patients with low-level expression (lower 25%) (Figure 6C). ln bladder cancers, the overall survival of patients with high mRNA expression (*P* = 0.0017 in CD98hc, and *P* = 0.0435 in LAT1) was lower than that of patients with low mRNA expression (Figure 6C).

CD98hc-independent cell-surface expression of LAT1 was suggested by the experiments using transfectants (Figure 1E). To confirm this, analyses using human cell lines were carried out. Anti-LAT1 mAb reacted with non-fixed living HEK293 and SW1116 colon cancer cells, whose CD98hc gene was almost completely disrupted by the CRISPR/Cas9-based KO (Figure 6D), suggesting that disulfide binding with CD98hc is not necessarily required for the cell-surface expression of LAT1. As shown in Figure 6E, 40-kDa LAT1 proteins were detected in HEK293, HeLa, HT29 and MIA PaCa-2 cells under reducing conditions, and the complexed LAT1-CD98 protein (125 kDa) was detected under non-reducing conditions. Of note, 40-kDa LAT1 was also detected in all cells under non-reducing conditions, suggesting that the association of the disulfide bond between human CD98hc and human LAT1 is not necessarily required for the membrane trafficking of LAT1, and that LAT1 monomers in addition to LAT1-CD98hc heterodimers exist on the cell surface.

## DISCUSSION

A relationship between CD98hc/LAT1 and oncogenesis has been suspected because CD98hc/LAT1 is coordinately overexpressed in human cancers (Figure 6A and Supplementary Figure 1). Furthermore, the relationship of CD98hc/LAT1 with cancer metastasis (Figure 6B) and poor prognosis (Figure 6C and Supplementary Figure 3) was demonstrated, therefore, CD98hc/LAT1 is strongly related to the malignancy of cancer cells. A marked positive correlation between higher expression of LAT1 in renal cancers and frequent metastasis/poor prognosis may explain the significant effects of mTOR inhibitors on renal cancers [45, 46]. In this context, LAT1 is required for efficient growth of KRAS-mutant colorectal cancer, and rapamycin reduces LAT1-deficient cell proliferation and tumor formation [47], suggesting LAT1 as an attractive therapeutic target for various cancers.

We demonstrated the oncogenicity of human CD98hc using the NIH/3T3 transformation system [21, 22], and the role of the association between introduced rat CD98hc and endogenous mouse CD98lc in the CD98-mediated transformation of Balb/3T3 cells [23]. In rat CD98hc, the 103rd cysteine residue (103C) is essential for disulfide bond formation with CD98 light chains, including LAT1 [23], based on the loss of oncogenicity of NIH/3T3 cells overexpressing mutant (103S) rat CD98hc. In humans, the 164th cysteine (164C) of LAT1 forms a disulfide bond with the 211th cysteine (211C) of CD98hc [48].

Many studies have reported anti-cancer effects due to the inhibition of LAT1 function [25, 44, 49]; however, the role of LAT1 overexpression in malignant transformation or carcinogenesis has not been clarified. In this study, we assessed the oncogenicity of LAT1 by the NIH/3T3 transformation system.

Overexpression of exogenous human LAT1 mRNA increased the mRNA level of endogenous mouse CD98hc in both naLAT1/3T3 and muLAT1/3T3 compared with coNIH/3T3 cells (Figure 1B), suggesting transcriptional regulation of CD98hc mRNA by LAT1. In this context, the downregulation of CD98hc protein by chicken LAT1 gene disruption [25] and siRNA-mediated knockdown [44] or CRISPR/Cas9-mediated KO [49] of human LAT1 resulted in the reduced protein expression of human CD98hc.

The present analysis of exogenous human LAT1 protein by WB revealed a similar amount of naLAT1 and muLAT1 proteins expressed in NIH/3T3 cell lines, and that both naLAT1 and muLAT1 proteins exist in membrane fractions, possibly embedded in endoplasmic or plasma membranes (Figure 1D). Furthermore, the reactivity of anti-LAT1 mAb against non-fixed living muLAT1/3T3 cells was stronger than that against naLAT1/3T3 cells (Figure 5A). This suggests that LAT1 can be expressed at the cell surface without associating with CD98hc, although past reports claimed CD98hc was required for the trafficking of LAT1 to the cell surface [5, 6]. This important biological characteristic of LAT1 was also substantiated by our present study, i.e., 40-kDa LAT1 proteins were detected under non-reducing conditions in WB analysis (Figure 1E and 6E), and anti-LAT1 mAb reacted with living HEK293 cells and SW1116 colon cancer cells whose CD98hc gene was completely disrupted by the CRISPR/Cas 9-based KO (Figure 6D). CD98hc-independent cell-surface expression of C98lc was not considered limited to LAT1 because anti-xCT mAb reacted with CD98hc-KO SW1116 cells (our unpublished data).

Regarding cell growth-related phenotypes, those of naLAT1/3T3 cells were significant. In particular, the phosphorylation of ERK, cell-cycle progression, and cell growth in the monolayer were promoted compared with coNIH/3T3 and muLAT1/3T3. The decrease in p27 in naLAT1/3T3 cells (Figure 2F) may have led to the altered proliferation of naLAT1/3T3 cells because upregulation of p27 and its inhibition of CDK2/cyclin E can downregulate cell growth.

LAT1 has been implicated in cancer growth and mTOR signaling. In this context, phosphorylation of 4EBP1 increased in naLAT1/3T3 compared with muLAT1/3T3 after BCAA stimulation (our unpublished data); therefore, BCAA, including leucine, may induce mTOR complex 1 (mTORC1)-mediated 4EBP phosphorylation.

Loss of contact inhibition and high saturation density are well-known indices for transformed or malignant cells. The morphology of these cells in the confluent state is depicted in Figure 2D. In naLAT1/3T3 cells, high-density status with local crisscrossed regions, which consists of spindle-shaped cells, was observed, although control NIH/3T3 and muLAT1/3T3 cells exhibited a well-aligned status with contact inhibition, as observed with original NIH/3T3 cells.

naLAT1/3T3 cells exhibited marked anchorage-dependent 3D growth in Matrigel and an increase in the number of pseudopods of colonies in naLAT1/3T3 cells was also noted (Figure 3A); therefore, the LAT1-CD98hc complex may be involved in the adhesion of cells to the extracellular matrix. In this context, the involvement of CD98 in regulating integrin affinity was previously reported [50].

Native (na) LAT1/3T3 cells also had stronger malignancy-related phenotypes (namely, *in vitro* anchorage-independent colony formation and *in vivo* tumor formation in nude mice) than control and mutant LAT1/3T3 cells, reflecting the probable oncogenicity of native LAT1, which can associate with CD98hc. Taken together, LAT1 may be required for the process of malignant transformation, with the condition of the association with CD98hc.

We previously developed 1st [42, 43] and 2nd generation [44] mAbs against human LAT1, and demonstrated their *in vitro* and *in vivo* anti-tumor effects on human cancer cells [25, 44]. Ab1 exerted *in vivo* anti-tumor effects on xenografted human cancer cells in immune-deficient mice, and *in vitro* effects, including internalization activity, inhibitory effects on amino acid uptake (most importantly, uptake of BCAA including leucine) and cellular growth, and antibody-dependent cellular cytotoxicity, were observed [44]. Ab1-mediated inhibition of leucine uptake may lead to the inhibition of mTOR signaling. Now, anti-LAT1 mAb also significantly inhibited *in vitro* cellular growth and *in vivo* tumor growth of naLAT1/3T3 cells, therefore, Ab1 may exert anti-tumor effects through mechanisms similar to those reported above.

Regarding the antigenicity or immunogenicity of LAT1 proteins, the binding of mutated LAT1 by anti-LAT1 mAb was superior to that of native LAT1. LAT1 is over-covered by CD98hc according to the recent structural analysis of LAT1 (48); therefore, possible LAT1 monomers in muLAT1/3T3 cells may be more easily accessed by anti-LAT1 mAb than LAT1 associated with CD98hc in naLAT1/3T3 cells. Related to this, we recently reported that the reactivity of anti-LAT1 mAbs increases in the presence of anti-CD98hc mAb [44], thus the association of CD98hc with LAT1 may downregulate the avidity of anti-LAT1 mAbs. As a related point, the serum level of anti-LAT1 mouse antibodies was higher in mice injected with muLAT1/3T3 cells than in those injected with naLAT1/3T3 cells (Figure 4C), demonstrating mutant LAT1 to be more immunogenic than native LAT1. This may lead to the development of more powerful anti-LAT1 therapeutic antibodies. Although existing molecular-targeting anti-cancer medicines are directed against receptor-type tyrosine kinases, differentiation antigens, angiogenesis-related molecules, and immune checkpoint molecules, the inhibition of cancer metabolism-related amino acid transport, especially the LAT1- transport system, is an attractive target and is expected to play a significant role in future cancer therapy.

## MATERIALS AND METHODS

### Animals

Mice were obtained from the Shimizu Animal Farm (Kyoto, Japan) and were maintained in the animal facility at Kindai University, and were maintained in specific pathogen-free conditions. They were housed individually in plastic cages under a standard light/dark cycle at a constant temperature of 23 ± 1°C. All experiments were approved by the Committee for the Care and Use of Laboratory Animals at Kindai University (KAPS-23-004).

### Cell culture

HeLa uterine, SW1116 and HT29 colorectal, and MIA PaCa-2 pancreatic cancer cell lines were purchased from American Type Cell Collection (ATCC, Manassas, VA, USA). These cell lines, NIH/3T3 (ATCC, CRL-165), human embryonic kidney (HEK) 293F (Invitrogen, Carlsbad, CA, USA), and HEK293 cells expressing LAT1 fused to green fluorescent protein (GFP) [42–44] were cultured in RD medium [44], which is a blended medium of equivalent volumes of DMEM and RPMI-1640 medium (Nissui Pharmaceutical Co., Ltd, Tokyo, Japan) with 7% heat-inactivated fetal bovine serum (FBS; #10270-106, Thermo Fisher Scientific Inc., Waltham, MA, USA) in a humidified CO_2_ (5%) incubator at 37°C. Aseptic processing was strictly controlled by a MediAir air purifier (Pieras Co., Ltd, Osaka, Japan).

### Establishment of NIH/3T3 cells expressing human native or mutant LAT1

NIH/3T3 cell lines expressing naLAT1/3T3 or muLAT1/3T3 were established using a pMYs-IRES-Puro retrovirus vector, which was kindly donated by Dr. Kitamura T (Institute of Medical Science, The University of Tokyo). NIH/3T3 cell lines (coNIH/3T3) transfected with the vacant pMYs-IRES-Puro retrovirus vector were also established. Cells were selected by 2 μg/mL of puromycin (Invitrogen) for more than 14 days and cloned by the limited dilution method.

### CD98hc-knockout (KO) cells

KO was performed as recently described [51, 52] using pX330 and pCAG-EGxxFP [53] purchased from Addgene (Watertown, MA, USA). For CRISPR/Cas9-based CD98hc (SLC3A2) gene disruption, guide (g) RNA sequences (5′-GCCGCGTTGTCGCGAGCTAC-3′) corresponding to the CD98hc gene (318-bp ~ 337-bp from the initiation ATG site) were designed using CRISPR direct (https://crispr.dbcls.jp/). The efficiency of KO by pX330 plasmids expressing codon-optimized SpCas9 and chimeric gRNA was confirmed by double-strand break-mediated enhanced GFP reconstitution with co-transfection of pX330 and pCAG-EGxxFP plasmids into HEK293 cells. Cells were seeded into 35-mm dishes (BD BioCoat, Franklin Lakes, NJ, USA) in 1 mL of RD medium, grown to 80% confluency, and plasmid DNA (5 μg) was introduced into cells using Xfect transfection reagent (Takara Bio Inc., Shiga, Japan). In the case of SW1116 cells, co-transfection of pX330 and pUC19 (#3219, Takara) containing the puromycin-resistant gene was carried out, and cells were cultured with puromycin (Invitrogen, 2 μg/mL) for 10 days.

### Reverse-transcription quantitative PCR (RT-qPCR)

RT-qPCR was performed as recently described [42]. Cells (3 × 10^5^) were dissolved in ISOGEN (Wako Pure Chemical) and incubated at 24°C for 5 min. Chloroform was added to the solution and incubated for 3 min. The sample was then centrifuged at 13,000 × g for 15 min. After isopropanol precipitation, the RNA pellet was rinsed gently with 70% ethanol and air-dried. The dried RNA pellet was dissolved in RNase-free water and RNA concentrations were measured spectrophotometrically at 260 nm. RT was performed using the Transcriptor First Strand cDNA Synthesis Kit (Roche Molecular Systems, Switzerland). RT-qPCR was performed with cDNA using gene-specific forward (*for*) and reverse (*rev*) primers (Sigma-Aldrich, Tokyo, Japan),

hLAT1, *for* 5′- GGCCGAGGAGAAGGAAGAGG-3′ and *rev* 5′- TGAGCTTCTGACACAGGACG-3′;

hCD98hc, *for* 5′- TGAGTTAGAGCCCGAGAAGC-3’ and *rev* 5′- TCCAGTTTCAGGCGTTCCAG-3′;

mLAT1, *for* 5′- CGGGCTGCCTGTCTACTTC-3′ and *rev* 5′- CAGAGCACCGTCACAGAGAA-3′;

mCD98hc, *for* 5′- TGTGGGAAAGCTGATGAATG-3′ and *rev* 5′- GACTCAGTCCCTGCAATCAAA-3′),

SYBR Green PCR Master Mix (Kapa Biosystems-Merck, Japan), and LightCycler 480 II (Roche) under the reaction conditions of 95°C for 10 min, followed by 35 cycles of heat denaturation at 95°C for 10 sec and annealing (55°C for 10 sec)/elongation (72°C for 4 sec).

### Antibodies

Ab1 anti-human LAT1 rat mAb [44], HR35 anti-human CD98 rat mAb [42~^44^], and MB872 anti-mouse CD98 rat mAb (23) were used. Polyclonal antibodies (pAbs) against CDK4 (sc-260), CDK2 (sc-163), P27 (sc-1641), and Cyclin E (sc-481) were purchased from Santa Cruz Biotechnology (Cosmo Bio Co., Ltd., Tokyo, Japan), and anti-Cyclin D1 (ab16663) pAb was obtained from Abcam (Tokyo, Japan). Rabbit pAbs against phospho-p44/42 MAPK (ERK1/2) (Thr202/Tyr204, #9101), p44/42 MAPK (ERK1/2, #9102), LAT1 (#5347), CD98hc (#47213), HER1/EGFR (#4267), and GAPDH (#2118) (Cell signaling technology, Danvers, MA, USA) were also used. Phycoerythrin (PE)-labelled anti-rat IgG (H+L) donkey pAb, PE-labelled anti-mouse IgG donkey pAb (#712-116-153, #715-116-151), horseradish peroxidase (HRP)-conjugated anti-rabbit IgG donkey pAb (#711-035-152), HRP-conjugated anti-mouse IgG donkey pAb (#715-035-151), and biotin-labelled anti-rat IgG donkey pAb (#712-066-150) were from Jackson ImmunoResearch (West Grove, PA, USA).

### Western blot (WB)

Cell lysates were prepared in lysis buffer (150 mM NaCl, 50 mM Tris (pH 7.4), 0.1% SDS, and 1% Nonidet P-40 (Nacalai Tesque), protease inhibitor cocktail (#03969-21, Nacalai), and phosphatase inhibitor cocktail (#07575-51, Nacalai) on ice for 15 min and then centrifuged at 20,000 × g for 10 min at 4°C. Proteins were extracted with the Subcellular Protein Fractionation Kit for Cultured Cells (#78840, Thermo Fisher, Tokyo). The cleared cell lysates were quantified using the BCA protein assay kit (#T9300A, Takara, Tokyo, Japan). Proteins (25 μg in each sample) in SDS sample buffer (45 mM Tris (pH 6.8), 1% SDS, 0.01% BPB, 10% glycerol, and 0.05 M DTT) were boiled at 95°C for 5 min and then separated by SDS-PAGE for 90 min. Separated proteins were transferred to Immobilon-P membranes (Millipore Japan, Tokyo) by semi-dry transfer-blot systems and blocked in 5% skim milk at 24°C for 1 h. The membranes were reacted with rabbit pAb or mouse mAb at 4°C for 16 h and incubated with HRP-conjugated anti-rabbit IgG or anti-mouse IgG 1: 10,000 diluted in phosphate-buffered saline (PBS) containing 0.05% Tween 20. The membranes were washed with PBS with 0.05% Tween 20 at each step and signals were detected with Chemi-Lumi One Super (Nacalai, #02230) by the Image Quant RT ECL Imager (GE Healthcare, Tokyo, Japan).

### Immunoprecipitation (IP)

Cells were treated with lysis buffer (150 mM NaCl, 50 mM Tris (pH7.4), 1% Nonidet P-40, and protease inhibitor cocktail (#03969-21, Nacalai) for 15 minutes at 4°C. Next, the supernatants were pre-cleared by protein G Sepharose 4 Fast Flow (50% slurry, GE Healthcare) at 4°C for 2 h and subjected to IP with anti-mouse CD98hc mAb (MB872, 20 μg) at 4°C for 12 h. Bead-bound proteins were collected by centrifugation at 9,000 × g for 1 min after mixing with protein G for 2 h. The beads were washed three times with lysis buffer (150 mM NaCl, 50 mM Tris (pH 7.4), and 1% Nonidet P-40) and with IP wash buffer (50 mM Tris, pH 8.0), and then suspended in DTT-free SDS sample buffer and heated at 95°C for 3 min. The supernatant separated by the centrifugation at 9,000 × g for 1 min was subjected to WB. The following procedures were performed as described in the paragraph of WB in Materials and Methods.

### Flow cytometry (FCM)

Cells (2 × 10^5^) were reacted with the primary mAbs (10 μg/mL) on ice for 1 h in each well of a 96-well plate (round bottom, Thermo Fisher / Nunc), followed by incubation with PE-conjugated anti-rat IgG secondary pAb (Jackson) for 45 min. Between each step, cells were washed with 0.2% bovine serum albumin (BSA, #01281-84, Nacalai)-PBS. Fluorescence intensity of the cell was measured by the LSR-Fortessa flow cytometer (BD, Franklin Lakes, NL, USA) and analyzed by FlowJo software (BD, Tokyo, Japan). Using the values of the mean fluorescence intensity (MFI) with or without the primary mAbs, the subtracted (Δ) MFI or the ratio (+ mAb/− mAb) of MFI (rMFI) was calculated.

### Cell growth and cell density analysis

NIH/3T3 cell lines (2 × 10^3^ cells in each well of 96-well plates) were suspended in 100 μl of RD medium supplemented with 7% FBS with or without Ab1 (10 μg/mL), and cultured at 37°C for 1–5 days in a CO_2_ incubator. WST-8-based CCK-8 reagent (Dojin Chemicals, Kumamoto, Japan) was added (5 μl/well) and absorbance at 450 nm was measured using a model 550 microplate reader (Bio-Rad, Tokyo, Japan). Cell density was analyzed by CKX53 microscope with a DP22-CUSW digital camera and CKX-CCSW software (Olympus, Osaka, Japan).

### Analysis of cell cycle progression

Cells were harvested by trypsinization, and washed with PBS by the centrifugation at 200 × g for 3 min. Cells were fixed with 70% ice-cold ethanol at –20°C, washed twice with 0.2% BSA-PBS, and then stained with 4′, 6-diamidino-2-phenylindole (DAPI, 1 μg/mL, Wako) at 4°C for 10 min. After washing cells with 0.2% BSA-PBS, FCM analysis was carried out.

### Anchorage-dependent growth in 3D culture

After Matrigel (Corning, NY, USA, 100 μl/well) was added to 8-well slide chamber slides (WATSON Bio Lab, Kobe, Japan), five hundred cells in 200 μL of medium were added to each well and cultured in a CO_2_ incubator at 37°C for 7 days. The number of colonies of greater than 50 μm in diameter was counted on images obtained by Biozero (Keyence, Osaka Japan).

### Anchorage-independent growth in 3D culture

One milliliter of 0.5% agarose (Lonza Japan) in RD medium was solidified in each well of a 6-well plate (Corning, NY, USA) as the bottom agarose layer and incubated for 20 min at 24°C. The 0.3% top agarose containing cells was poured over the 0.5% agarose gel (2,500 cells/well) and set at 10 min at 4°C. The plate was cultured in a humidified incubator at 37°C with 5% CO_2_ for 14 days, rinsed twice with PBS, and fixed with methanol for 15 min. Colonies were stained for 20 min with Giemsa’s solution (Merck, Darmstadt, Germany) diluted 1: 20 in phosphate buffer (4.7 mM KH_2_PO_4_, 2 mM Na_2_HPO_4_). Digital images of the colonies were taken using a Biozero microscope (Keyence). Colonies were counted using Image J software (NIH, USA).

### Tumor formation of NIH/3T3 cells overexpressing LAT1 in nude mice

Male KSN nude mice at 6 weeks of age were subcutaneously inoculated with transfectants (1 × 10^6^). After confirmation of visible tumors, tumor volumes were measured every 2 days using digital calipers. In the additional experiment, anti-LAT1 mAb (Ab1) (100 μg in 500 μl PBS) or control mAb was *i.p.* administered to nude mice with tumors that were inoculated with cells (4 × 10^6^), followed by an additional injection of the same amount of mAb on days 7 and 14. The tumor volume (mm^3^) was calculated by the formula 0.5 × (length) × (width)^2^.

### Scatchard plot analysis

The avidity of Ab1 against naNAT1/3T3 or muLAT1/3T3, which express native or mutant LAT1, was evaluated by Scatchard plot analysis [44, 54]. Cells were reacted with different concentrations (1 ng ~ 30 μg/mL) of mAb for 1 h on ice. Following removal of unbound mAbs by washing with 0.2% BSA-PBS, cells were incubated with PE-conjugated anti-rat IgG secondary pAb on ice for 45 min and then analyzed by FCM. From the values of MFI with or without the primary mAbs, the subtracted (Δ) MFI was calculated. ΔMFI/mAb concentrations were plotted against the ΔMFI, and the dissociation constant: K_D_ (nmol/L) and avidity constant: K_A_ (M^-1^) were determined from the slope of linear regression.

### Statistical analysis

All data are shown as the average ± SEM. The criteria for significance were ^*^ *P* < 0.05, ^**^ *P* < 0.01, and ^***^ *P* < 0.001. The data were analyzed by one- or two-way analysis of variance (ANOVA) followed by Tukey’s post hoc multiple comparison test (Figures 1–5).

## SUPPLEMENTARY MATERIALS



## Abbreviations

BCAA: branched-chain amino acids
BSA: bovine serum albumin
FBS: fetal bovine serum
FCM: flow cytometry
GFP: green fluorescent protein
IP: immunoprecipitation
LAT1: L-type amino acid transporter 1
mAb: monoclonal antibody
mTOR: mammalian target of rapamycin
pAb: polyclonal antibody
PBS: phosphate-buffered saline
SLC: solute carrier
TCGA: The Cancer Genome Atlas
WB: Western blot
