# Supplementary material for: Oncogenic transformation of NIH/3T3 cells by the overexpression of L-type amino acid transporter 1, a promising anti-cancer target
Source: Oncotarget. 2021 Jun 22;12(13):1256–70. doi: 10.18632/oncotarget.27981 (PMC8238248; doi:10.18632/oncotarget.27981)
Supplement: Supplementary file 1 [file oncotarget-12-1256-s001.pdf]

# Oncogenic transformation of NIH/3T3 cells by the overexpression of L-type amino acid transporter 1, a promising anti-cancer target

## SUPPLEMENTARY MATERIALS

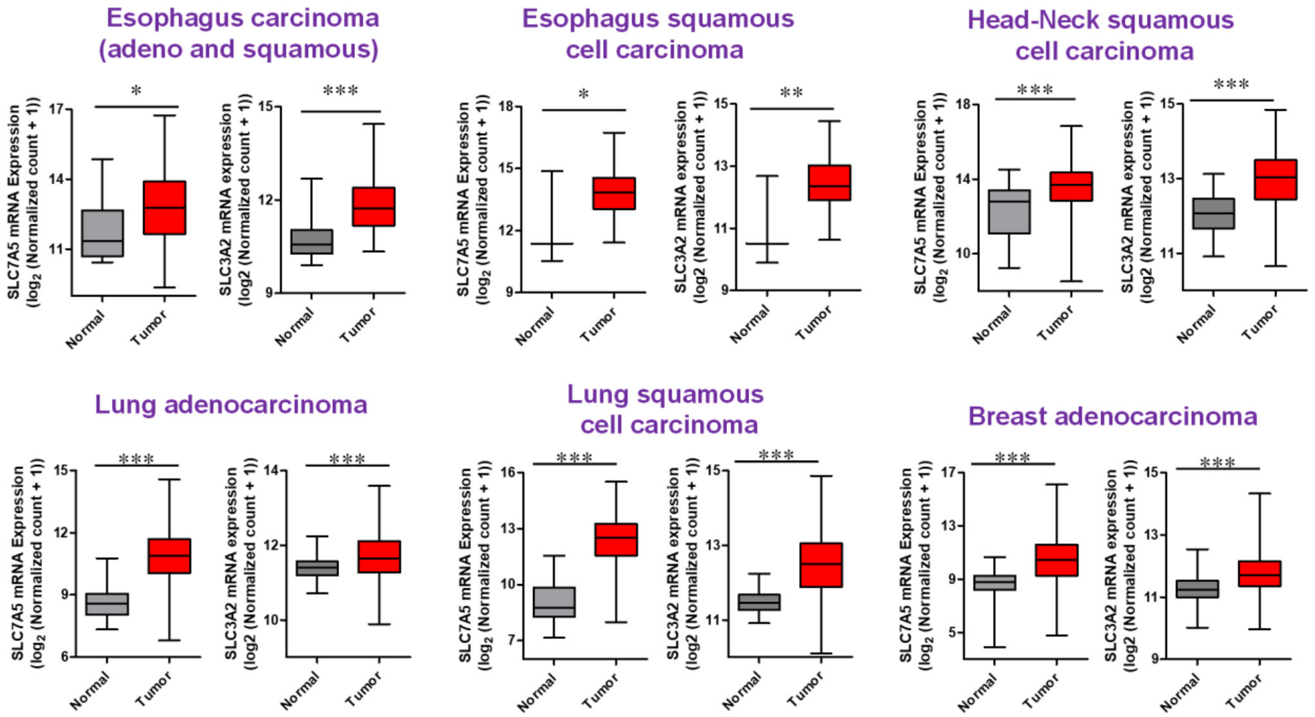


**Supplementary Figure 1: Coordinated higher expression of SLC7A5 (LAT1) and SLC3A2 (CD98hc) in various epithelial cancers.** Box-whisker plot of the expression of SLC7A5 (left) / SLC3A2 (right) in normal and tumor tissues. *P*-values were calculated by the Student's *t* test.

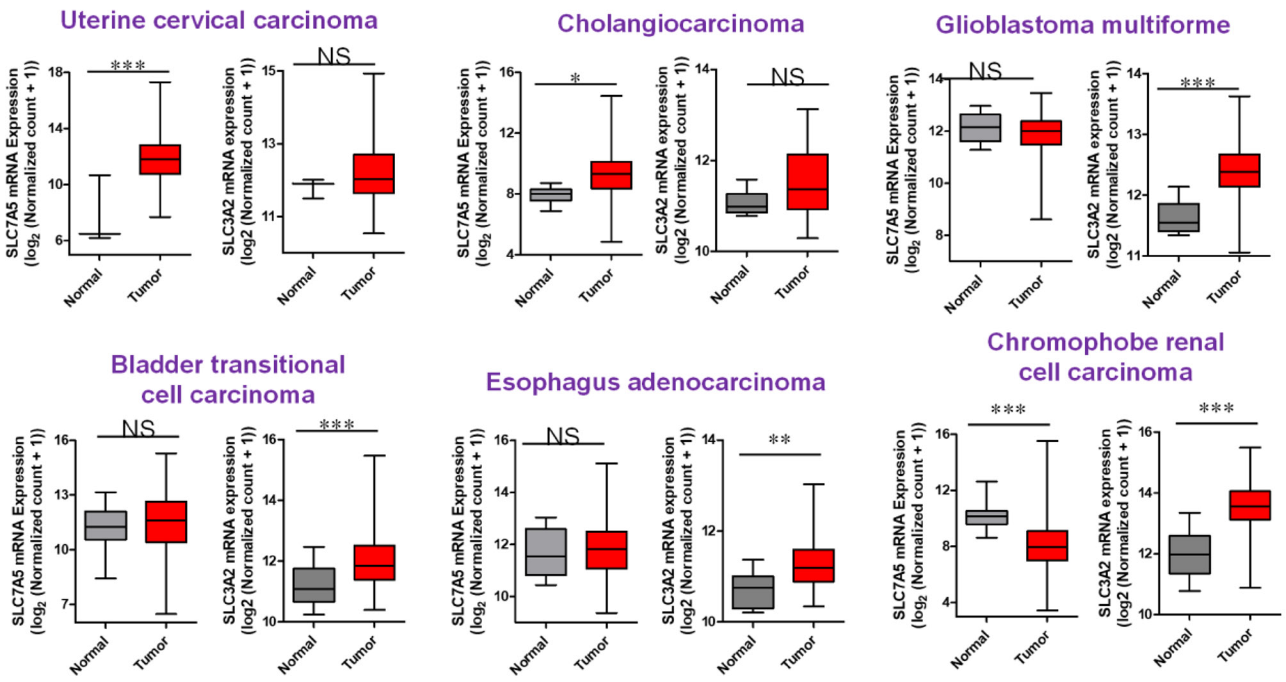


**Supplementary Figure 2: Higher expression of SLC7A5 (LAT1) in uterine cervical carcinoma and cholangiocarcinoma, and higher expression of SLC3A2 (CD98hc) in glioblastoma, bladder carcinoma, esophagus adenocarcinoma and chromophobe renal cell carcinoma.** Box-whisker plot of the expression of SLC7A5 (left) / SLC3A2 (right) in normal and tumor tissues. *P*-values were calculated by the Student's *t* test.

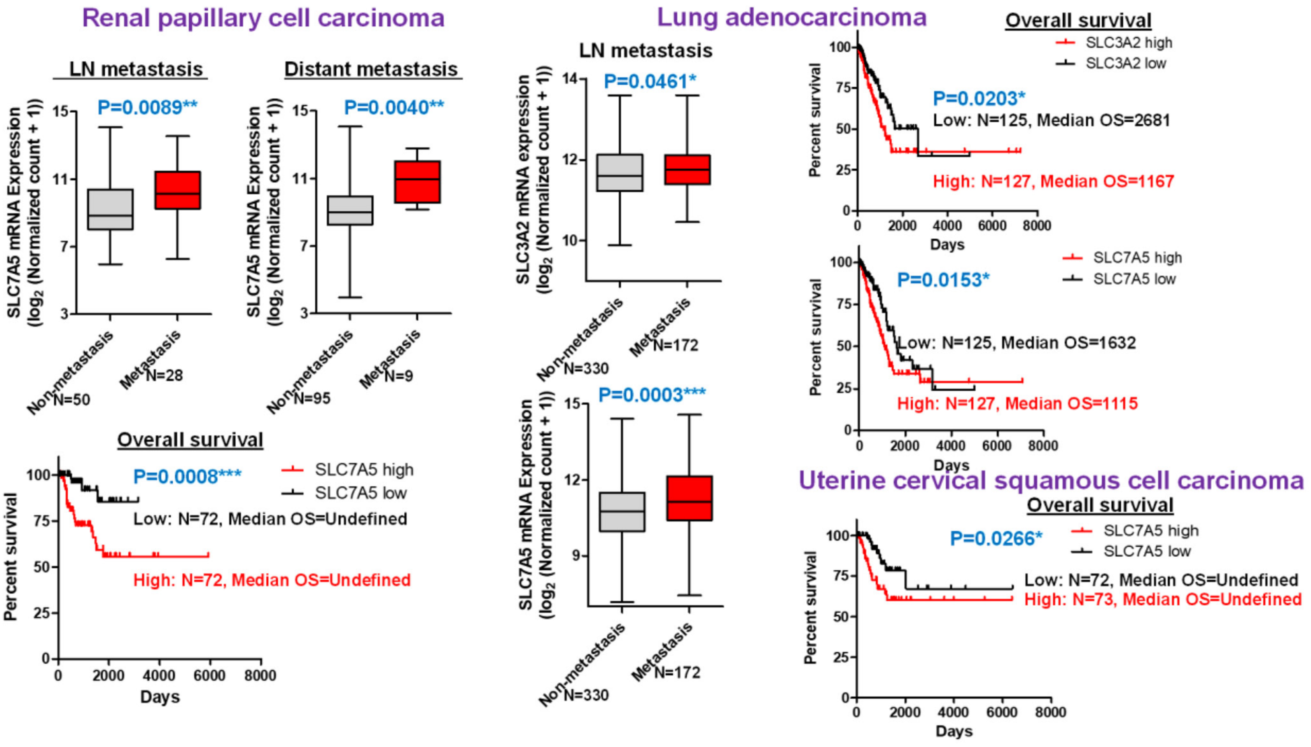


**Supplementary Figure 3: The positive correlation between SLC7A5 (LAT1) expression and metastasis (LN and distant) and overall survival in renal papillary cell carcinoma, that between SLC3A2 (CD98hc)/SLC7A5 expression and LN metastasis and overall survival in lung adenocarcinoma, and that between SLC7A5 expression and overall survival in uterine cervical carcinoma were evaluated.** Kaplan-Meier survival analysis with a log rank test was carried out. The correlation between mRNA expression (top 25% and lower 25%) and survival was analyzed.
